# Chronic *Kirschsteiniothelia* infection superimposed on a pre-existing non-infectious bursitis of the ankle: the first case report of human infection

**DOI:** 10.1186/s12879-018-3152-3

**Published:** 2018-05-22

**Authors:** Masanori Nishi, Ichiro Okano, Takatoshi Sawada, Yasuka Hara, Kiwamu Nakamura, Katsunori Inagaki, Takashi Yaguchi

**Affiliations:** 1 Department of OrthopaedicSurgery, Ohta-Nishinouchi Hospital, 2-5-20 Nishinouchi, Koriyama, Fukushima 963-8558 Japan; 2 Department of Respiratory Medicine, Ohta-Nishinouchi Hospital, 2-5-20 Nishinouchi, Koriyama, Fukushima 963-8558 Japan; 3 Department of Infection Control, Fukushima Medical University, 1 Hikarigaoka, Fukushima, Fukushima 960-1295 Japan; 4 Department of Orthopaedic Surgery, Showa University School of Medicine, 1-5-8 Hatanodai, Shinagawa-ku, Tokyo 142-8666 Japan; 5 Medical Mycology Research Center, Chiba University, 1-8-1 Inohana, Chuo-ku, Chiba, Chiba 260-8673 Japan

**Keywords:** *Kirschsteiniothelia*, Bursitis, Black fungus, Human infection

## Abstract

**Background:**

*Kirschsteiniothelia* is a saprophytic fungus that is abundantly present in the environment. To date, there have been no reports of human infection caused by this fungus. We report a case of *Kirschsteiniothelia* infection superimposed on a pre-existing non-infectious bursitis of the ankle.

**Case presentation:**

An 81-year-old immunocompetent female local farmer noticed the presence of a nodule on her right ankle 5 years before her first visit to our hospital. A cystic mass of approximately 45 mm × 30 mm was present at the tip of the right lateral malleolus. Culture of the aspirated fluid revealed visibly black colonies and characteristic blackish hyphae; nucleotide sequence of the internal transcribed spacer region was determined and compared in a GenBank database. The results indicated *Kirschsteiniothelia* infection.

**Conclusions:**

We described the first case of *Kirschsteiniothelia* infection manifested as ankle bursitis. The disease seemed to be localized and systemic antibiotics had not been used in this case. However, continued observation is needed because of the possibility of disease progression with the pathogen.

**Electronic supplementary material:**

The online version of this article (10.1186/s12879-018-3152-3) contains supplementary material, which is available to authorized users.

## Background

Black fungus, or dematiaceous fungus, is a generic term used to describe fungi whose developing colonies exhibit a distinct black to grayish color by producing dark melanoid pigments on the cell walls of vegetative cells and/or conidia [1]. Black fungi are abundant in the environment, and over 60 genera and 300 species of black fungi exhibit pathogenicity to humans and animals [2]. However, *Kirschsteiniothelia*, known as a phytopathogenic fungus and the teleomorphic form of *Dendryphiopsis*, has never been reported to cause infections in humans. Here, we report the first confirmed case of *Kirschsteiniothelia* soft-tissue infection in humans.

## Case presentation

**Fig. 1:**
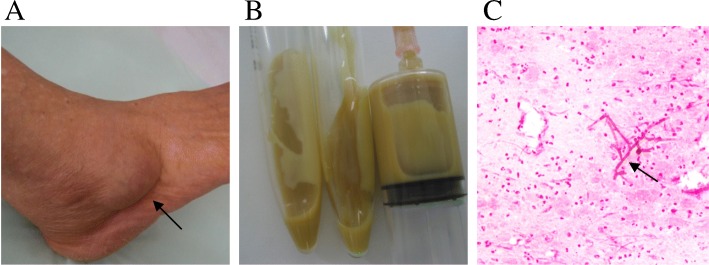
Clinical pictures. **a** An image of the right ankle showing a 30 × 45 mm nodule, **b** Aspiration of the nodule showing yellowish-white fluid, **c** Gram stain of the fluid showing accumulated neutrophils and Gram-negative branching hyphae (magnification of × 200)

**Fig. 2:**
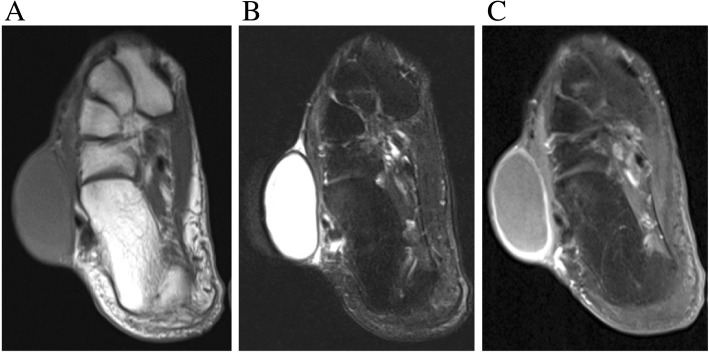
Magnetic resonance imaging. **a** T1-weighted image showing iso-intensity mass, **b** T2-weighted image indicating high-intensity mass, **c** T1-weighted image with gadolinium contrast showing strong marginal enhancement

An 81-year-old female local farmer living in a rural region of northeast Japan noticed a nodule on her right ankle 5 years before her first visit to our hospital. She had a history of hypertension, hyperlipidemia, and osteoporosis, but had no history of trauma to her right ankle. The patient did not seek immediate medical attention, and the nodule gradually increased in size, after which she visited our hospital. A physical examination revealed a cystic mass of approximately 45 × 30 mm at the tip of the right lateral malleolus (Fig. 1a). There was no tenderness, redness, or local heat. The patient had no functional impairment of her right ankle joint. Needle aspiration of the mass revealed accumulation of approximately 10 mL of yellowish white homogeneous fluid (Fig. 1b). Gram stain of the fluid revealed accumulated neutrophils and Gram-negative branching hyphae (Fig. 1c). X-ray images revealed osteoarthritis of the ankle joint. Magnetic resonance imaging (MRI) scan with contrast revealed a well-defined T1 iso−/T2 high-intensity mass with strong marginal enhancement on contrast imaging (Fig. 2). White blood cell count and serum C-reactive protein level were within the normal limit. Microscopic analysis of the fluid revealed no organism or crystal, like monosodium urate and calcium pyrophosphate. Culture of the aspirated fluid showed negative findings. After the initial visit, the patient discontinued the follow-up visits.

**Fig. 3:**
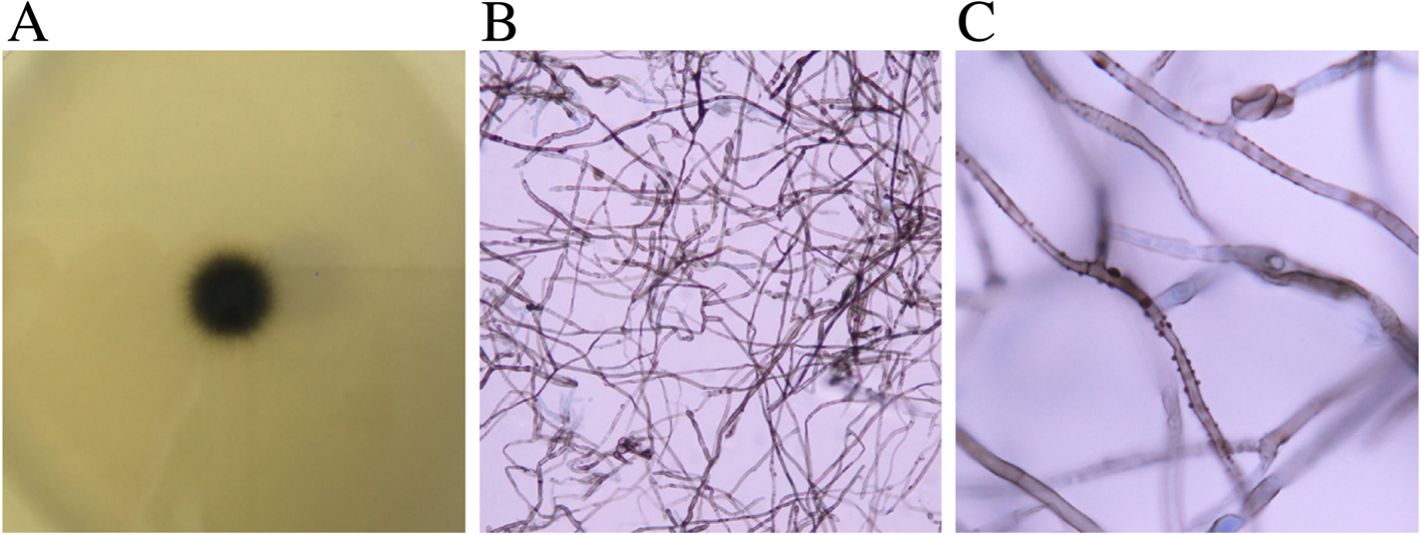
Microbiological examination of the pathogen. **a** Picture of the *Kirschsteiniothelia* growing on Sabouraud dextrose agar, **b** Microscopic imaging of the fungus (magnification of × 400), **c** Microscopic imaging of the fungus (magnification of × 1000)

One year after the initial visit, the patient returned to our hospital because of pain in the right ankle joint. The mass did not appear to have changed in size. Aspiration of the fluid was repeated with no change in its characteristics. However, culture of the aspirated fluid revealed growth of black colonies (Fig. 3a). Microscopic analysis detected blackish hyphae (Fig. 3b, c). The fungus was classified as a branching group of *Kirschsteiniothelia* by sequencing the internal transcribed spacer (ITS) regions. A second aspiration performed 1 month later showed the same results on morphological and molecular biological examinations. The sensitivity patterns of antifungal agents were studied (Table 1). Fluid accumulation recurred shortly after needle aspiration; however, the patient became otherwise asymptomatic and refused further treatment, such as antimycotics or surgical debridement; the patient is currently under careful observation.

**Table 1:** Results of the antifungal susceptibility test

Antifungal agent	MICs (μg/mL)
Micafungin	0.5
Caspofungin	4
Amphotericin B	> 16
Flucytocine	0.12
Fluconazole	16
Itraconazole	> 8
Voriconazole	> 8
Miconazole	1

*MIC* minimum inhibitory concentration

### Microbiological examination

Growth of black colonies was obtained on Sabouraud dextrose agar (Purmedia^®^, Eiken Chemical Co., Ltd., Tokyo, Japan) after 10 days of incubation at 25 °C (Fig. 3a), and no growth was observed on blood agar/bromothymol blue lactose agar (TSA^®^, Nippon Becton Dickinson Company, Ltd., Tokyo, Japan). Microscopic analysis revealed 2.5–3.0 μm, brown to black, septate, smooth, or verrucose hyphae with dark brown droplets, which were blanching (Fig. 3b, c). There was no conidia or spore formation that would lead to species identification.

### Molecular examination

The fungal DNA was extracted using PrepMan-Ultra Sample Preparation Reagent^®^ (Applied Biosystems, Foster City, CA) from the culture on potato dextrose agar (Difco™ Potato Dextrose Agar, Nippon Becton Dickinson Company, Ltd.) after 7 days of incubation at 25 °C. The ITS regions of fungal ribosomal DNA were amplified with primers ITS4 and ITS1 [3]. The sequence of the ITS region was determined using ABI Prism 3130 N^®^ (Applied Biosystems, Foster City, CA) (Additional file 1) and compared with other known species in the GenBank database using the Basic Local Alignment Search Tool (BLAST) algorithm [4] (Additional file 2). BLAST searches demonstrated 89–91% identity matches with *Kirschsteiniothelia* and *Dendryphiopsis* species in the *Kirschsteiniotheliaceae* family (91%, *K. aethiops*, Genbank no. AF377283.1; 91%, *Dendryphiopsis atra,* Genbank no. HF677175.1; 90%, *K. lignicola*, Genbank no. HQ441567.1; and 90%, *K. emarceis*, Genbank no. NR_138375.1).

## Discussion and conclusions

Black fungi are saprophytic in nature and mainly exist in soil, decaying plants, and trees worldwide. The typical form of black fungus infection is called chromoblastomycosis, which is characterized by chronic, progressive, cutaneous, and subcutaneous infection caused by black fungi. The most commonly infected organ in humans is the skin. The fungi usually invade the body through minor wounds; hence, the lesions are mostly found in the exposed parts of the body. Infection in other internal organs, such as brain abscesses, were also reported mostly among immunocompromised patients [5], but are extremely rare in immunocompetent patients. Most patients with cutaneous black fungal infection are farmers, as was our patient [6], probably due to occupational exposure to plants and soil [7].

Three reports of black fungal bursitis were found in the English literature [8–10] (Table 2). A patient with olecranon bursitis had a long history of post-traumatic non-infectious bursitis at the olecranon, and the fungus infected the pre-existing fluid accumulated in the bursa. In our case, the microscopic analysis of fluid aspirated at her first visit showed negative findings. There are two possible explanations for this: the patient had only non-infectious bursitis at that time, or the causal fungus existed but was not cultured due to technical reasons. As the clinical course and symptoms of our patient at the first visit were compatible with non-infectious bursitis, we believe a superimposed infection was highly likely to have occurred in our case. All reported cases were in immunocompromised hosts due to chronic medical conditions who needed treatment with surgical debridement and antifungal medications. There was no report on black fungal bursitis among immunocompetent patients. The immune condition of our patient may have resulted in less severe symptoms and a long clinical course.

**Table 2:** Summary of reported cases of black fungal bursitis

Reference	Sex/age	Major co-morbidities	Infection Site	Pathogen	Treatment	Country
Padhye et al. (1995) [9]	F/72	Wegener’s granulomatosis (Prednisone, methotrexate)	Knee	*Mycoleptodiscus indicus*	Antifungal	USA
Linas et al. (2005) [8]	F/54	MDS IgAD	Olecranon	*Phaeoacremonium* spp.	Excision, antifungal	USA
Almagro-Molto et al. (2016) [10]	F/55	Post-kidney-pancreas transplantations (Prednisone, tacrolimus)	Knee	*Rousoella percutanea*	Antifungal	Germany
Nishi et al. (Presenting)	F/81	None	Ankle	*Kirschsteiniothelia* spp.	Aspiration	Japan

*Kirschsteiniothelia* was first identified from the class *Dothideomycetes* by Hawksworth et al. in 1985 [11]. Boonmee et al. revised its taxonomy and introduced the *Kirschsteiniotheliaceae* family using nucleotide sequence analysis in 2012 [12]. By 2017, 22 species of the *Kirschsteiniotheliaceae* family had been identified [13]. In Japan, Hosoya et al. reported that a species of the *Kirschsteiniothelia* genus was identified on wood in Yakushima Island (*K. incrustans*) [14]. *Kirschsteiniothelia* has been known to infect plants and is found in decaying trees, soil, and water worldwide, similar to several other black fungi; however, *Kirschsteiniothelia* infections in humans or animals have never been reported. In our case, repeated cultures of the fluid aspirate detected black colonies and blackish hyphae, which is consistent with the characteristics of other *Kirschsteiniothelia* spp. [12, 15–18]. Later the diagnosis was confirmed by comparison analysis of the nucleotide sequence of the ITS region. Currently, molecular identification is an essential tool for the diagnoses of rare fungal infections, along with classical morphological identification; it has also contributed to the revision of the taxonomy of fungi [19], including *Kirschsteiniothelia* [12], as well as the discovery of novel species. Da Cunha et al. compared the DNA sequences of 101 clinical isolates of morphologically identified *Curvularia* spp. and reported that the species could not be confidently identified in over 25% of the isolates. The authors suggested that some isolates belonged to putative new species [20]. In our case, we could not obtain a perfect species match with the nucleotide sequence. Although not all known *Kirschsteiniothelia* species have been deposited in the GenBank database, it is possible that the causal fungus is a new species of *Kirschsteiniothelia*.

For treatment of black fungal infections, no standard therapy has been established, but previous studies demonstrated that voriconazole, posaconazole, and itraconazole presented in vitro activity against these infections [1]. Neither the standard assay of drug sensitivity nor its break point has been established for *Kirschsteiniothelia*. However, the pathogenic fungus did not appear to be inhibited in vitro with high concentrations of itraconazole and voriconazole (posaconazole was not examined); this implies that these agents may not be effective against *Kirschsteiniothelia* infection. In our case, no treatment was performed except aspiration of the accumulated fluid; therefore, we could not assess the in vivo efficacy of antifungal drugs. Further studies are needed to guide the indication and efficacy of antifungal drugs for *Kirschsteiniothelia* infection in humans.

Finally, most black fungus infections occur in immunocompromised hosts. We believe that the patient requires long-term observation because it is possible that similar symptoms may recur or that invasive disease may manifest in future if the patient’s immune status deteriorates.

In conclusion, this is the first case report of human infection by *Kirschsteiniothelia*. The diagnosis was confirmed by repeated morphological and molecular analyses. This patient received no treatment except fluid aspiration; thus, long-term observation is mandatory and further studies are needed to establish the treatment strategy for this fungal infection.

## Additional files

Additional file 1: Nucleoid sequence of the internal transcribed spacer region. (PPTX 45 kb)

Additional file 2: The result of molecular analysis with GenBank database using the Basic Local Alignment Search Tool (BLAST) algorithm (lcl|Query_85557 is the specimen from the presenting patient). (PDF 100 kb)

## Abbreviations

BLAST: Basic Local Alignment Search Tool
ITS: the internal transcribed spacer
MRI: Magnetic Resonance Imaging
