# Supplementary figures and images for: Chronic Kirschsteiniothelia infection superimposed on a pre-existing non-infectious bursitis of the ankle: the first case report of human infection
Source: BMC Infect Dis. 2018 May 22;18:236. doi: 10.1186/s12879-018-3152-3 (PMC5964637; doi:10.1186/s12879-018-3152-3)

## Slide 1
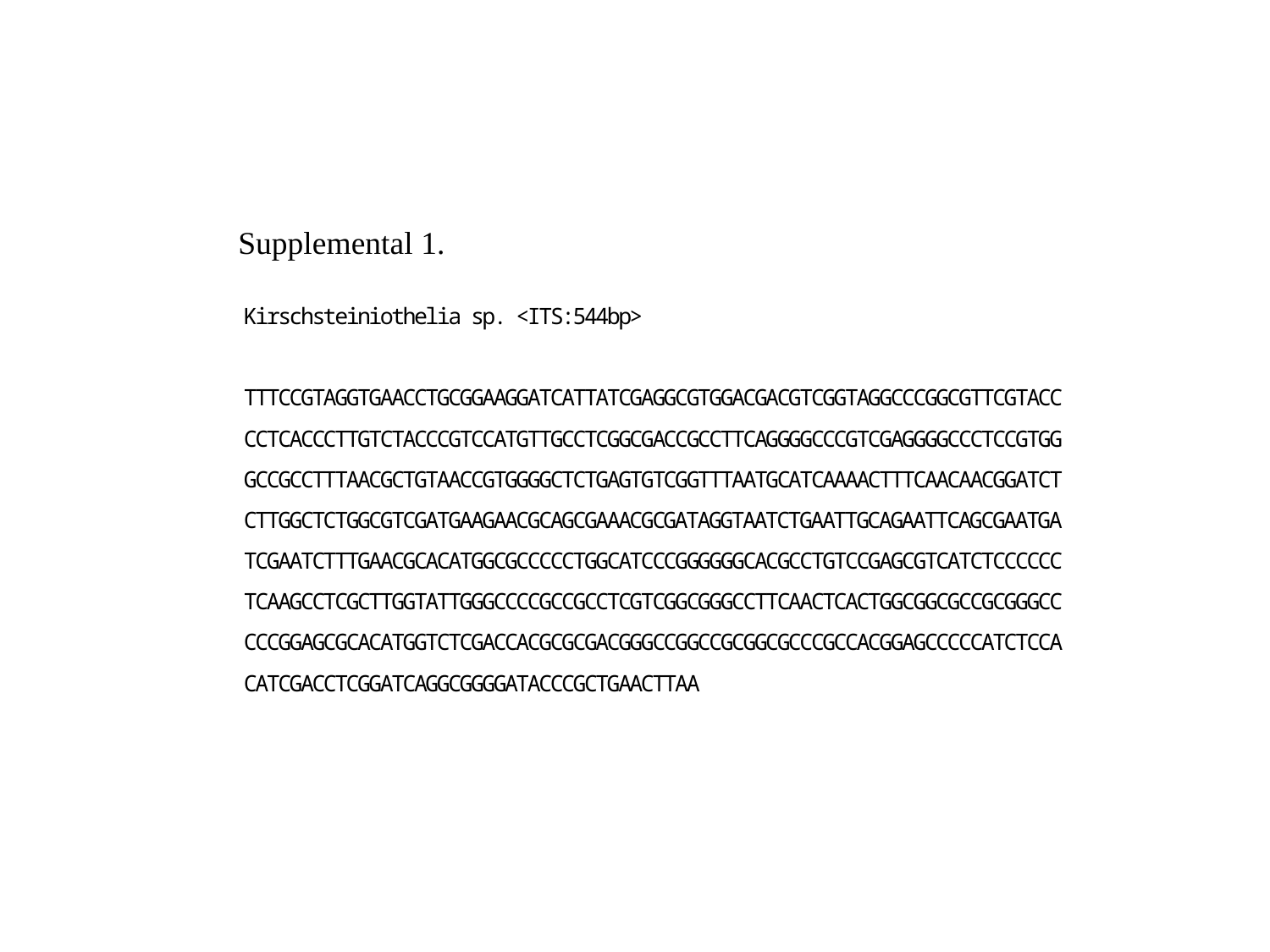

Supplemental 1.

Supplement: Supplementary file 1 — Nucleoid sequence of the internal transcribed spacer region. (PPTX 45 kb) [file 12879_2018_3152_MOESM1_ESM.pptx]

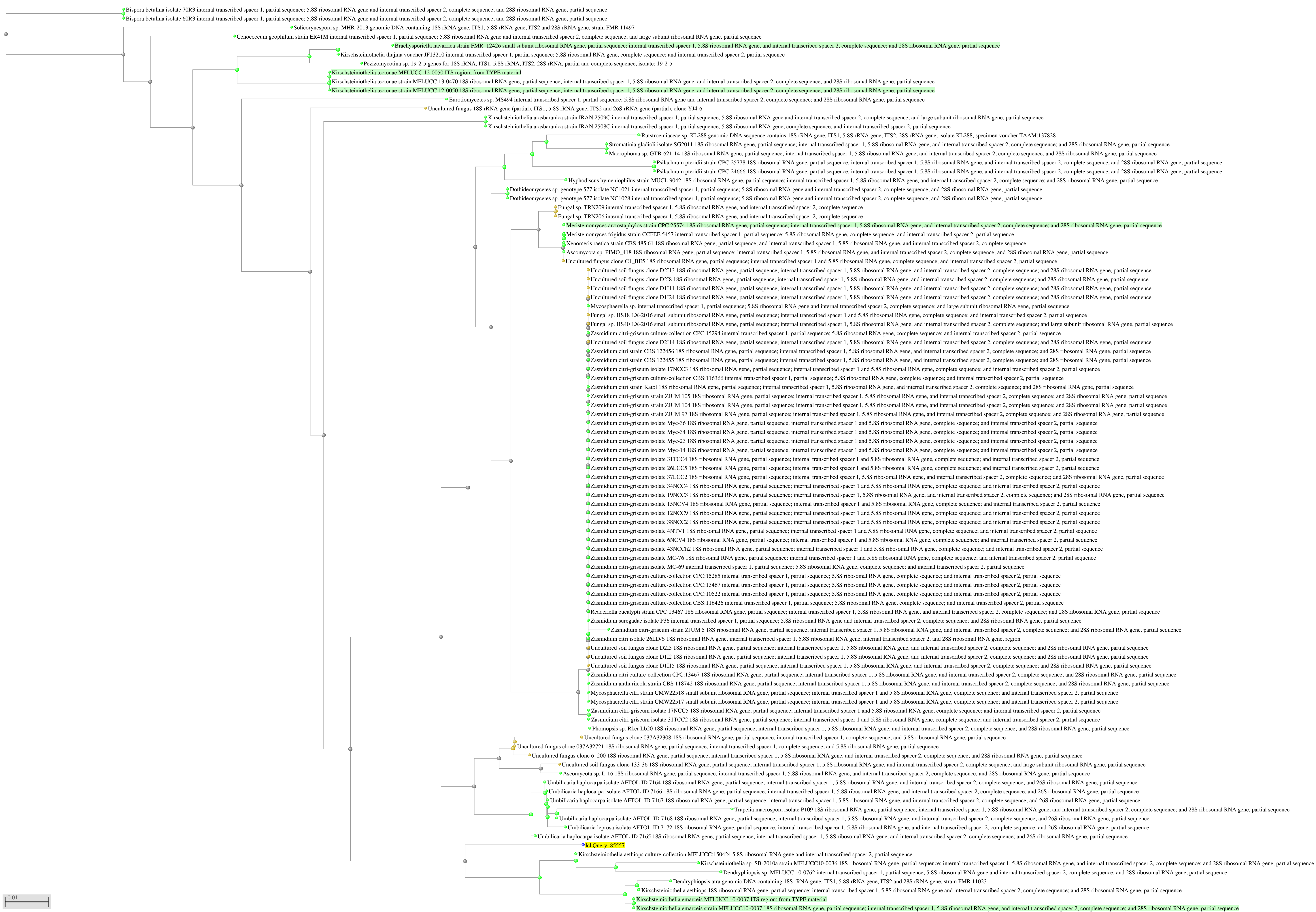

Supplement: Supplementary file 2 — The result of molecular analysis with GenBank database using the Basic Local Alignment Search Tool (BLAST) algorithm (lcl|Query_85557 is the specimen from the presenting patient). (PDF 100 kb) [file 12879_2018_3152_MOESM2_ESM.pdf]
